# Vet To Vet Maine: COVID 19 Effect on a Veteran Companion Pilot Study in Reducing Social Isolation and Loneliness

**DOI:** 10.1093/geroni/igab046.2753

**Published:** 2021-12-17

**Authors:** Marilyn Gugliucci, Amy Lin, Shirley Weaver

**Affiliations:** 1 University of New England College of Osteopathic Medicine, Kennebunk, Maine, United States; 2 University of New England, University of New England, Maine, United States; 3 University of New England College, University of New England, Maine, United States

## Abstract

Background: Maine veterans represent 11.8% of Maine population, twice that of the United States. Veterans are at risk for social isolation, loneliness and suicide. The mission of Vet To Vet (V2V) Maine, a non-profit organization, connects trained veteran volunteers with fellow veterans (veteran friends) for companionship, assistance with benefits, and support independent living. This study: (1) assessed if V2V program fulfilled its mission; and (2) determined any care partner effects from the program. Methods: Mixed methods research spanned 6-months, 2019-2020. Twenty-four participants; trained Veteran Volunteers (VV) were paired with Veteran Friends. Four Care Partners (CP) of Veteran Friends participated. Assessments included pre/post Veteran Rand Health Survey (VR-12), Patient Health Questionnaire (PHQ-9), and Late Onset Stress Symptomatology (LOSS) Short Form. CPs completed pre/post Zarit Burden Interview (ZBI-22) assessments. Qualitative interviews focused on visits/activities, relationship building, and program feedback. Data analyses included Wilcoxon Sign Test and NVivo 12+ Qualitative Data Analysis Software. Results: Pre/post data failed to show significance (P=.05), however trends supported an improvement in mental and physical health scores. COVID-19 was a confounding variable as state stay-at-home orders occurred at the companion program study mid-point. Three key themes included; (1) Veteran Companionship; (2) Effects of COVID; and (3) Care Partners. The V2V Companion program was determined effective and reported highly successful relationship matches. CPs confirmed the importance and benefits of V2V. Conclusion: The V2V Maine companion program pilot research supported success in connecting Veterans Volunteer and Veteran Friends; fostering companionship, friendships, mentoring, assisting with benefits, and supporting independent living.

